# Potential Effects of Anthocyanin-Rich Roselle (*Hibiscus sabdariffa* L.) Extract on the Growth, Intestinal Histomorphology, Blood Biochemical Parameters, and the Immune Status of Broiler Chickens

**DOI:** 10.3390/antiox11030544

**Published:** 2022-03-13

**Authors:** Shimaa A. Amer, Hanan S. Al-Khalaifah, Ahmed Gouda, Ali Osman, Nehal I. A. Goda, Haiam A. Mohammed, Mahmoud I. M. Darwish, Aziza M. Hassan, Sherif Kh. A. Mohamed

**Affiliations:** 1 Department of Nutrition and Clinical Nutrition, Faculty of Veterinary Medicine, Zagazig University, Zagazig 44511, Egypt; 2 Environment and Life Sciences Research Center, Kuwait Institute for Scientific Research, P.O. Box 24885, Safat 13109, Kuwait; hkhalifa@kisr.edu.kw; 3 Animal Production Department, Agricultural & Biological Research Division, National Research Center, Dokki, Cairo 11865, Egypt; ag.abdullah@nrc.sci.eg; 4 Biochemistry Department, Faculty of Agriculture, Zagazig University, Zagazig 44511, Egypt; aokhalil@zu.edu.eg; 5 Department of Histology and Cytology, Faculty of Veterinary Medicine, Zagazig University, Zagazig 44511, Egypt; nehalgoda@zu.edu.eg; 6 Department of Physiology, Faculty of Veterinary Medicine, Zagazig University, Zagazig 44511, Egypt; haawadallah@vet.zu.edu.eg; 7 Department of Biochemistry, Faculty of Veterinary Medicine, Zagazig University, Zagazig 44511, Egypt; midarwish@zu.edu.eg; 8 Department of Biotechnology, College of Science, Taif University, P.O. Box 11099, Taif 21944, Saudi Arabia; a.hasn@tu.edu.sa; 9 Department of Anatomy and Embryology, Faculty of Veterinary Medicine, Zagazig University, Zagazig 44511, Egypt; skmohamed@vet.zu.edu.eg

**Keywords:** broilers, anthocyanin, growth, intestinal morphometry, bird’s health

## Abstract

The potential effects of anthocyanin-rich roselle, *Hibiscus sabdariffa* L. extract (ARRE) on the growth, carcass traits, intestinal histomorphology, breast muscle composition, blood biochemical parameters, antioxidant activity, and immune status of broiler chickens were evaluated. In the present study, *Hibiscus* acidified ethanolic extract was reported to have a total anthocyanin content of about 359.3 mg cyanidin 3-glucoside/100 g DW, total polyphenol concentration (TPC) of about 598 mg gallic acid equivalent (GAE)/100 g DW, and total flavonoids (TFs) of about 100 mg quercetin equivalent (QE)/100 g DW. Two-hundred-fifty one-day-old chicks (Ross 308 broiler) (87.85 gm ± 0.32) were randomly allotted to five experimental groups and fed on basal diets supplemented with five levels of ARRE: 0, 50, 100, 200, and 400 mg Kg^−1^ for 35 days. Dietary ARRE addition did not improve the birds’ growth and carcass traits. Supplemental ARRE increased the n-3 polyunsaturated fatty acids (PUFA) (ω-3) percentage in the breast muscle. Dietary ARRE increased the villous height, and the ARRE100 group raised the villus height to crypt depth ratio. Dietary ARRE increased the immunoexpression of immunoglobulin G (IgG) in the spleen. The serum thyroxine hormone (T4) level was higher in the ARRE200 group. The serum growth hormone level was increased by ARRE addition in a level-dependent manner. According to the broken-line regression analysis, the optimum inclusion level of ARRE was 280 mg Kg^−1^. All levels of supplemental ARRE decreased the serum triglyceride level. The serum total antioxidant capacity (TAC) was increased in the ARRE100-ARRE400 groups, the serum superoxide dismutase (SOD) level was increased in the ARRE200 group, and the serum malondialdehyde (MDA) level was decreased by increasing the ARRE level. Supplemental ARRE significantly increased the serum levels of lysozymes and IL10. The serum complement 3 (C3) level was increased in ARRE200 and ARRE400 groups. It can be concluded that dietary ARRE addition had many beneficial effects represented by the improvements in the bird’s metabolic functions, blood biochemistry, intestinal morphology, antioxidant activity, immune status, and higher ω-3 content in the breast muscles. However, it had no improving effect on the birds’ growth.

## 1. Introduction

Poultry production is one of the most quickly developing protein sources worldwide, which has become very important to worldwide food sources. Recent poultry feeds are manufactured to satisfy their nutritional needs. Still, more research is required to determine whether these feeds sufficiently maintain the birds’ immunity system or if additional components are needed to enhance their immune function [1]. Furthermore, the growth performance improvements in broiler chickens are associated with intestinal health [2,3,4,5]. Using antibiotics as growth promoters are prohibited because of the rise of multiple and cross-resistance antibiotics used in treating human and animal infections [6]. Earlier studies demonstrated that phytobiotics with antiviral, antimicrobial, fungicidal, anticoccidial, and antioxidant properties could be used as antibiotic alternatives in poultry diets [7,8].

*Hibiscus sabdariffa* L. (Roselle) is traditionally used in herbal beverages as a food, in the food industry as a flavoring agent, and as an herbal medication. Its extracts exhibited antioxidant, antibacterial, hepato- and nephroprotective, diuretic effect, hypolipidemic, antihypertensive, antidiabetic effects. These effects may be related to intense antioxidant activities, inhibition of α-glucosidase and α-amylase, direct vasorelaxant effect, inhibition of angiotensin-converting enzymes (ACE), and modulation of calcium channel. Roselle has an excellent record of safety and tolerability [9]. The pharmacological actions of roselle are related to its component such as flavonoids, anthocyanins, organic acids, and polysaccharides [10]. Roselle extracts contain a high proportion of organic acids as hibiscus acid, hydroxycitric acid, citric acid, tartaric and malic acids as leading composites, and ascorbic and oxalic acid as small compounds [11].

Anthocyanins are glycosidic forms of anthocyanidins that give fruits and flowers bright and attractive colors, and they form a subgroup of flavonoids [12]. Anthocyanins have several properties in humans and animals, such as anti-inflammatory, antimicrobial, antioxidant activities. They prevent oxidative liver damage and hyperglycemia, prevent cardiovascular diseases and diabetes, improve vision and obesity control [13,14,15]. Moreover, anthocyanins prevent atherosclerosis, reduce free radical activity, and decrease inflammation and aging [16]. Therefore, anthocyanin can be used as a suitable feed additive. Many studies demonstrated the anthocyanin effects on different animal species [17,18,19,20]. Dietary anthocyanins may enhance intestinal barrier function, improve host/bacteria bonding, and boost the multiplication of beneficial bacteria such as *Lactobacillus* spp. and *Bifidobacterium* spp. [21]. These bacteria benefit host health through antimicrobial effects on pathogenic microorganisms [22,23].

The only edible supplies of anthocyanins are fruits and vegetables [24,25]. The anthocyanins levels in fruits are considerably greater than in vegetables [26]. The highest anthocyanin content was recorded in some berries such as blueberry, huckleberry, chokeberry, and cranberry, while the lowest anthocyanin content was recorded for grapefruit, date, and figs. The richest vegetables in anthocyanins and anthocyanidins are purple potato, purple cabbage, and red cabbage [27]. However, the total anthocyanin content in fruits and vegetables differs greatly between different genera and cultivars and is greatly influenced by temperature, light, and agronomic factors [28].

Anthocyanins are currently used as feed constituents due to their potential antioxidant and immunostimulant activities. So far, their impacts on poultry are less known [29,30]. Hence, this study investigated the possible effects of anthocyanin-rich roselle extract in broiler chicken diets on the growth, intestinal morphology, blood biochemical parameters, fatty acid composition of breast muscles, antioxidant activity, and immune status.

## 2. Materials and Methods

### 2.1. Preparation and Description of the Anthocyanin-Rich Roselle Extract

#### 2.1.1. Anthocyanin-Rich Roselle Extract Preparation

Dehydrated roselle calyces were obtained from the local market, Zagazig City, Sharqia Governorate, Egypt. Dehydrated roselle calyces were ground into a powder with milling; then, the ethanolic extract was prepared according to Zhao et al. [31]. Then, 10 g of flour was blended with 100 mL acidified ethanol (70%) solution (0.1 M HCl (*v*/*v*)). These combinations were agitated at 200 rpm for 24 h at room temperature in the dark before being filtered using Whatman No. 42 filter paper (Whatman^®^ quantitative filter paper, ashless, Grade 42, Merck KGaA, Darmstadt, Germany). Ethanol was separated from the extract using vacuum in a BüCHI B-480 water bath evaporator (Marshall Scientific, Cambridge, MA, USA) at 45°C, then lyophilized in a freeze drier (Thermo-electron Corporation–Heto power dry LL 300 Freeze dryer).

#### 2.1.2. Determination of Bioactive Compounds in Anthocyanin-Rich Roselle Extract

Total anthocyanin concentration was estimated using pH differential protocol [32] as described in Wu et al. [33]. The absorbance (*Ab*) was calculated by Equation (1).

(1) Ab=(Ab510−Ab700)pH1.0−(Ab510−Ab700)pH4.5

Total anthocyanin concentration was calculated from the Equation (2), and the results were expressed as mg of cyanidin 3-glucoside/100 g.

(2) Total anthocyanin concentration =(Ab/e∗L)∗MW∗(D/G)∗V∗100

where *Ab* is absorbance, e is the molar extinction coefficient of cyanidin 3-glucoside (26,900), *L* is the cell length (1 cm), *MW* is anthocyanins molecular weight (449.2), *D* is dilution factor, *V* is the final volume (mL), and *G* is the dry weight (DW) of roselle flour (mg).

The Folin–Ciocalteu test was used to assess total phenolic content (TPC). The standard curve was made with gallic acid. TPC was measured in mg of gallic acid equivalent (GAE)/100 gm of dry material [34]. The calibration equation for gallic acid (Equation (3)) was:

(3) y=0.001x+0.0563 (R2=0.9792)

where *y* and *x* are the gallic acid absorbance and concentration in µg/mL, respectively.

The total flavonoids (TFs) were measured according to the procedure outlined before [35]. The standard curve was created using quercetin, with total flavonoid concentration expressed as quercetin equivalent (QE). Total flavonoids contents were stated as quercetin equivalent (QE), which was calculated based on the calibration curve (Equation (4)).

(4) y=0.0012x+0.008 (R2=0.944)

where y is the absorbance and x is the concentration of quercetin in µg/mL.

#### 2.1.3. Roselle Anthocyanin Estimation by HPLC

The high-performance liquid chromatography (HPLC) analysis was conducted according to Durst and Wrolstad [36]. Freeze-dried extract in the amount of 10 mg was dissolved with 5 mL of methanol. The Sample was centrifuged at 5000× *g* for 5 min, and the supernatant was collected and filtered through a Millipore membrane (0.45 µm). The filtrate was twice diluted with purified distilled water. The analyses were performed on an HPLC (Agilent, Santa Clara, CA, USA) model-LC 1100 series, equipped with a degasser, an autosampler automatic injector, a high-pressure pump, and a UV/Visible detector at multiple wavelengths wave. HPLC experiments were conducted using a reversed-phase C18 column (Prontosil, 250 × 4.0 mm, 5 µm, (Bischoff, Roseville, CA, USA). The mobile phase used was a binary gradient eluent (solvent A, 0.1% trifluoroacetic acid in water; solvent B, 0.1% trifluoroacetic acid in acetonitrile). Acetonitrile used was of HPLC grade (Sigma/Aldrich, Burlington, MA, USA) and was degassed in an ultrasonic bath before use. The water was distilled using a Milli-Q system (Millipore, Sigma/Aldrich, Burlington, MA, USA). The elution program was 5–20% B (0–5 min), 20–35% B (5–10 min), 35–100% B (10–25 min), and 100% B (25–40 min) with a flow rate of 0.8 mL·min^−1^. The chromatograms were monitored at 521 nm.

Anthocyanin’s identification and peak assignments are based on their retention durations, UV–VIS spectra comparisons, and published data. The cyanidin 3-*O*-galactoside was used to measure anthocyanin levels.

### 2.2. Birds, Experimental Design, and Diets

This research was conducted in a poultry research unit in the faculty of veterinary medicine, Zagazig University, Egypt, to assess the effect of dietary supplementation of different levels of anthocyanin-rich roselle extract (*Hibiscus sabdariffa* L.) (ARRE) on growth performance, intestinal histomorphology, immune status, antioxidant activity, the fatty acid profile of breast muscles, and blood biochemical parameters of broiler chickens. All experiment procedures were approved by the Institutional Animal Care and Use Committee (ZU-IACUC) of Zagazig University, Egypt (Approval No. ZU-IACUC/2/F/17/2022).

In total, 250 1-day-old chicks (Ross 308 broiler) were obtained from a commercial chick producer. Before starting the experiment, birds were submitted to a 3-day adaptation period to reach an average body weight of 87.85 gm ± 0.32**.** Then they were randomly allotted to five experimental groups with five replicates for each (10 chicks/replicate). Birds were fed on basal diets supplemented with five levels of ARRE: 0, 50, 100, 200, and 400 mg Kg^−1^ for 35 days. The proximate chemical composition of the basal diet is shown in (Table 1). The managerial conditions and the experimental diets were conducted following Ross 308 broiler nutrition specifications AVIAGEN [37].

### 2.3. Growth Performance

The birds were individually weighed on the fourth day of age to obtain the average initial body weight; then, the body weight was recorded at 10, 23, and 35 days to calculate the average body weight of the birds in each group.

The body weight gain (BWG) was calculated by Equation (5).

(5) BWG=W2−W1

where W2 is the final body weight at the intended period, and W1 is the initial body weight in the same period.

Feed intake (FI) of each replicate was recorded as the difference between the weight of the feed offered and residues left and then divided by the number of birds in each replicate to find out the average feed intake per bird.

The feed conversion ratio (FCR) was calculated by Equation (6).

(6) $$FCR=\frac{FI\;\left(g\right)}{BWG\;\left(g\right)}$$

The relative growth rate (RGR) was calculated using Equation (7) described by [38].

(7) $$RGR=\frac{W2-W1}{0.5\left(W1+W2\right)}\times 100$$

where W1 = the initial live weight (g), W2 = the live weight at the end of the considered period (g).

Protein efficiency ratio (PER) was determined by Equation (8) according to [39].

(8) $$PER=\frac{Live\;weight\;gain\;\left(g\right)\;}{protein\;intake\;\left(g\right)}$$

### 2.4. Carcass Traits

At the end of the experiment, ten birds from each treatment were chosen for carcass traits evaluation, according to Amer et al. [2].

### 2.5. Determination of the Chemical and Fatty Acid Composition of the Breast Muscle

At the end of the experiment, breast muscle samples (5 samples/group) were taken. Oils from the breast muscle were extracted using a solvent mixture of chloroform/methanol (2:1, *v*/*v*) [40]. Fatty acids in the extracted oil and the chemical composition of the breast muscle (dry matter, fat, crude protein, ash content %) were determined according to AOAC [41].

### 2.6. Sample Collection and Laboratory Analyses

At the end of the feeding period, blood samples (two aliquots) were randomly collected after slaughter (two birds/replicate, ten birds/group). The chicks were euthanized using cervical dislocation, according to the American Veterinary Medical Association guidelines [42]. The first aliquot of blood was placed in tubes containing dipotassium salt of Ethylene diamine tetra acetic acid (EDTA )as an anticoagulant for hematological analysis by Hemascreen 18 Automatic Cell Counter (Hospitex Diagnostics, Sesto Fiorentino, Italy) according to Harrison et al. [43]. The differential leukocytes count was estimated as Schalm et al. [44] described. The second aliquot of blood was collected without anticoagulant, left to clot at room temperature, centrifuged for 15 min at 3500 rpm for serum separation, and stored at −20 °C in deep freezing until biochemical analysis. Samples from different parts of the small intestine were taken for histomorphology examination. Spleen samples were taken for immunohistochemistry.

#### 2.6.1. Blood Biochemical Indices

Chicken ELISA kits (My Biosource Co. San Diego, CA, USA) of CAT. NO. MBS269454, MBS265796, MBS025331, and MBS266317 were used for Triiodothyronine (T3) and Thyroxine (T4), leptin, and growth hormones determination, respectively, following the instructions of the enclosed pamphlets of each kit.

The serum levels of glucose, creatinine, and uric acid were measured by an automatic biochemical analyzer (Robotnik Prietest ECO Ambernath (W), Thane, India) [45,46,47]. The method of Reitman and Frankel [48] was used to estimate serum levels of aspartate aminotransferase (AST) and alanine aminotransferase (ALT).

#### 2.6.2. Serum Lipid Profile and Proteinogram

Colorimetric diagnostic kits of spectrum-bioscience (Egyptian Company for Biotechnology, Cairo, Egypt) were used for measuring the serum total cholesterol (TC), triglycerides (TG), and high-density lipoprotein cholesterol (HDL-C), following the methods of Allain et al. [49], McGowan et al. [50], and Vassault et al. [51], respectively. The low-density lipoprotein cholesterol (LDL-C) level was calculated following the Iranian formula LDL-C = TC/1.19 + TG/1.9 − HDL/1.1 − 38. The very low-density lipoprotein cholesterol (VLDL-C) was measured using the turbidimetric method described by Griffin and Whitehead [52].

The serum level of total protein was determined according to Grant [53]. The serum albumin level was evaluated according to Doumas et al. [54]. The serum globulin level was calculated mathematically by subtracting albumin values from total proteins [55].

#### 2.6.3. Antioxidant Activity

The serum total antioxidant capacity (TAC) was estimated as mentioned by Rice-Evans and Miller [56], catalase (CAT) was calculated according to Aebi [57], superoxide dismutase (SOD) activity was evaluated according to Nishikimi et al. [58], and the Malondialdehyde (MDA) level was determined according to Mcdonald and Hultin [59].

#### 2.6.4. Immune Indices

The serum level of interleukin 10 (IL10) was determined using chicken ELISA kits of MyBioSource Co. of CAT.NO. MBS701683. Meanwhile, the serum complement 3 level was determined using a sandwich enzyme-linked immunosorbent assay (ELISA) kit (Life Span Biosciences, Inc., Seattle, WA, USA) of CAT. NO. LS-F9287). The serum lysozyme activity was determined according to Lie et al. [60].

### 2.7. Histological Examination of the Small Intestine

Two-centimeter samples (3 samples/group) were taken from each part of the small intestine (the duodenum, jejunum, and ileum) and preserved in 10% neutral buffered formaldehyde (NBF) for 72 h, then processed for dehydration and clearing, and embedded in wax. Histological study was performed on 5 µm thick transverse sections (cut by a microtome), fixed on slides, and stained with hematoxylin and eosin [61]. The villous height (VH) was measured from the tip (with a lamina propria) of the villus to the base (villus-crypt junction), and the crypt depth (CD) was calculated from the villus-crypt junction to the distal limit of the crypt.

### 2.8. Immunohistochemical Procedures

At the end of the experiment, spleen samples (3 samples/group) were collected for examination of immunoexpression of immunoglobulin G (IgG) according to Saber et al. [62]. Briefly, samples were directly trimmed and immersed in neutral buffer formalin. Fixation of samples was conducted for four days. Routine histological techniques were performed on all the samples, including the previous steps used in histological sections such as dehydration, clearance, embedding, and cutting by microtome. Tissue ribbon was mounted on positively charged slides to avoid separation during the autoclaving step. Then slides were rehydrated through immersion in xylene, alcohols, and water. The antigen retrieval step aimed to remove methylene bridges on the protein caused by formalin. Therefore, it is too essential to unmask the antigen epitopes to allow the antibodies to bind. This step was carried out by immersion of the samples in a solution of 0.05 M citrate buffer, pH 6.8. Inhibition of the endogenous cellular enzymes to avoid nonspecific binding of horseradish peroxidase (HRP) or alkaline phosphatase (AP). Thus, samples were put in 0.3% H_2_O_2_ and protein block with sera of the animal spp. of the secondary antibody at room temperature for 30 min. After that, slides were incubated with a goat anti-Chicken IgG (Cat. No. NBP1-72720, Novus Biologicals, Briarwood Avenue, USA). The slides were rinsed with PBS three times for 10 of each. Slides were visualized with a DAB kit (3,3′-Diaminobenzidine) and eventually stained with Mayer’s hematoxylin as a counterstain. The staining intensity was assessed by positive areas per area using ImageJ ecosystem (IJ 1.46r, 2012, National institutes of health NIH, WA, USA), and data were expressed as the percent of positive area. Labeling indices were performed by counting positive cells in 1000 cells.

### 2.9. Statistical Analysis

Data were analyzed with a one-way analysis of variance (ANOVA) using the GLM procedure in SPSS (SPSS Inc., Chicago, Illinois, USA) after Shapiro–Wilk test was used to verify the normality and Levene’s test was used to verify homogeneity of variance components between experimental treatments. Tukey’s test was used to compare the differences between the means at 5% probability. Variation in the data was expressed as pooled SEM, and the significance level was set at *p* < 0.05. The broken-line regression with Tukey’s test considered information on BWG, FCR, growth hormone, and thyroxin hormone for determining the optimum supplementation level of ARRE.

## 3. Results

### 3.1. Anthocyanin Description

**Figure 1:**
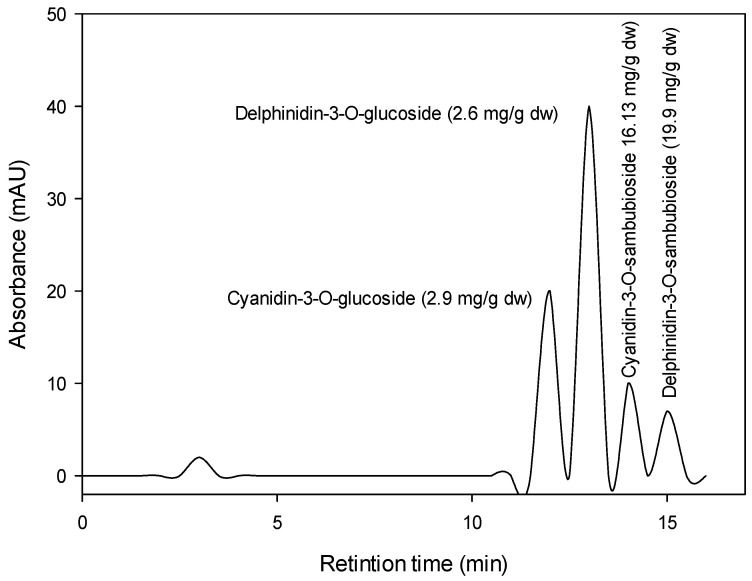
HPLC chromatogram of *Hibiscus* anthocyanins at 521 nm.

The *Hibiscus* acidified ethanolic extract was reported to have total anthocyanin content (TAC) of about 359.3 mg cyanidin 3-glucoside/100 g DW, total polyphenol concentration (TPC) of about 598 mg gallic acid equivalent (GAE)/100 g DW, and total flavonoids (TFs) of about 100 mg quercetin equivalent (QE)/100 g DW. Figure 1 shows the chromatographic analysis of *Hibiscus* anthocyanins at 521 nm. Four anthocyanins (Cyanidin-3-*O*-glucoside, Delphinidin-3-*O*-glucoside, Cyanidin-3-*O*-sambubioside, and Delphinidin-3-*O*-sambubioside) were recorded in the *Hibiscus* acidified ethanolic extract. The most prevalent anthocyanins were Delphinidin-3-*O*-sambubioside (19.9 mg/g DW) and Cyanidin-3-*O*-sambubioside (16.13 mg/g DW).

### 3.2. Growth Performance

Table 2 shows the effect of supplemental ARRE on the growth parameters of broiler chickens. During the starter period, there was a linear decrease in the feed intake and FCR in the ARRE400 group compared with the ARRE0 group (*p* < 0.05), while the BW and BWG were not significantly different between the groups (*p* > 0.05). During the grower period, there was a linear decrease in the BW and BWG in the ARRE400 group (*p* < 0.05), while the FI and FCR were not significantly different between the groups (*p* > 0.05). Dietary addition of ARRE had no significant effect on the growth performance of birds during the finisher period and on the allover performance (*p* > 0.05).

### 3.3. Carcass Traits

As shown in Table 3, ARRE addition had no significant effect on the weights of carcass, intestine, gizzard, liver, and lymphoid organs (spleen and bursa) relative to the live weight (*p* > 0.05).

### 3.4. Chemical and Fatty acid Composition of the Breast Muscle

Dietary ARRE addition had no significant effect on the dry matter, fat, crude protein, and ash content of the breast muscle (*p* > 0.05). The n-3 PUFA (ω-3) percentage and the ω-3 to n-6 PUFA (ω-6) ratio were linearly increased in the breast muscle of broiler chickens by supplemental ARRE in a level-dependent manner (*p* < 0.01). At the same time, the ω-6 percentage was not significantly different between groups (*p* > 0.05) (Table 4).

### 3.5. Morphometric Measures of the Small Intestine

**Figure 2:**
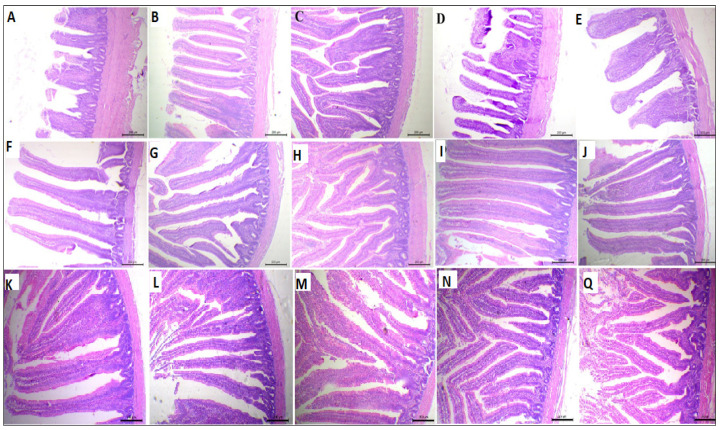
Photomicrograph of the small intestine: duodenum (**A**–**E**), jejunum (**F**–**J**), and ilium (**K**–**Q**) stained with hematoxylin and eosin stain; ARRE0 (**A**,**F**,**K**), ARRE50 (**B**,**G**,**L**), ARRE100 (**C**,**H**,**M**), ARRE200 (**D**,**I**,**N**), ARRE400 (**E**,**J**,**Q**).

The Effect of supplemental ARRE on the morphometric measures of the small intestine is shown in Table 5 and Figure 2. The VH was quadratically increased in the ARRE50 and ARRE100 groups (*p <* 0.01). The duodenal villous height to crypt depth ratio (VH: CD) and goblet cell count (GCC) were quadratically higher in the ARRE100 group compared with other groups (*p <* 0.01). The jejunal VH was quadratically raised in the ARRE100 group (*p <* 0.01). The GCC in the jejunum was linearly and quadratically increased in all ARRE supplemented groups (*p < 0.05*) except for the ARRE400 group, which was not significantly different from the control group (ARRE0). The highest jejunal GCC was observed in the ARRE100 group. The VH and CD of the ileum were quadratically increased in the ARRE50 and ARRE100 groups (*p* *<* 0.05). The GCC of the ileum was linearly and quadratically increased in all ARRE supplemented groups (*p <* 0.01) compared with the ARRE0 group. The highest GCC was observed in the ARRE100 group. The VW of the ileum was not significantly different among the groups (*p >* 0.05) except for the ARRE400 group that was linearly decreased (*p =* 0.01) in comparison with the control group. The VH: CD in the jejunum and ileum was not significantly different between all treatments (*p >* 0.05).

### 3.6. Immunohistochemical Analysis

**Figure 3:**
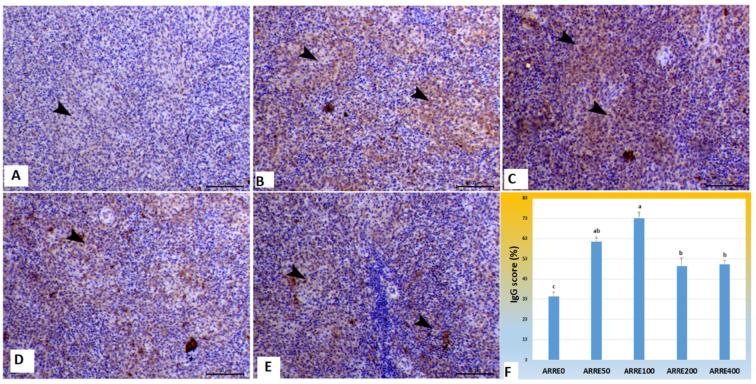
Photomicrograph of splenic tissues immunostained with IgG antibody: (**A**) spleen of ARRE0 group showing mild expression of IgG (31.42%) within white pulp (arrowhead); (**B**) The spleen of the ARRE50 group showed an increase of IgG immunostaining expression of IgG (58.49%) within white pulp (arrowheads); (**C**) The spleen of ARRE100 showed a marked increase of IgG immunostaining (70.25%) within the white pulp (arrowheads); (**D**) The spleen of ARRE200 showed an increase of immunostaining expression of IgG (46.40%) within the white pulp (arrowheads); (**E**) The spleen of ARRE400 showed increased IgG immunostaining (47.41%) within white pulp (arrowheads). Bar = 50 µm. (**F**) showed morphometric measures of IgG immunostaining expression (%). ^a,b^ means carrying different superscripts are significantly different at (*p <* 0.05).

The immunoexpression of IgG in the spleen is illustrated in Figure 3. Immunostained spleen sections treated with specific monoclonal IgG antibodies demonstrated increased staining reactions within the white pulp in all experimental groups 58.49, 70.25, 46.40, 47.41% for ARRE50, ARRE100, and ARRE200, ARRE400, respectively, with the highest reaction observed in the ARRE100 group compared with mild expression in the control group (31.42%).

### 3.7. Blood Hematology

The effect of supplemental ARRE on blood hematology is shown in Table 6. The red blood cells (RBCs) count was quadratically increased in the ARRE100 group (*p* < 0.01). The platelets count was linearly and quadratically decreased in ARRE100 and ARRE 200 groups (*p <* 0.05). The mean corpuscular volume (MCV) value was not significantly different in all groups (*p* > 0.05) except for the ARRE100 group, which was quadratically decreased compared with the control group (*p* < 0.01). The mean corpuscular hemoglobin (MCH) value was not significantly different in all groups (*p* > 0.05) except for the ARRE50 group, which was quadratically decreased compared with the control group (*p* < 0.01). The highest heterophils and eosinophils counts were observed in ARRE400 and ARRE50, respectively (*p* < 0.05).

### 3.8. Serum Biochemical Parameters

**Figure 4:**
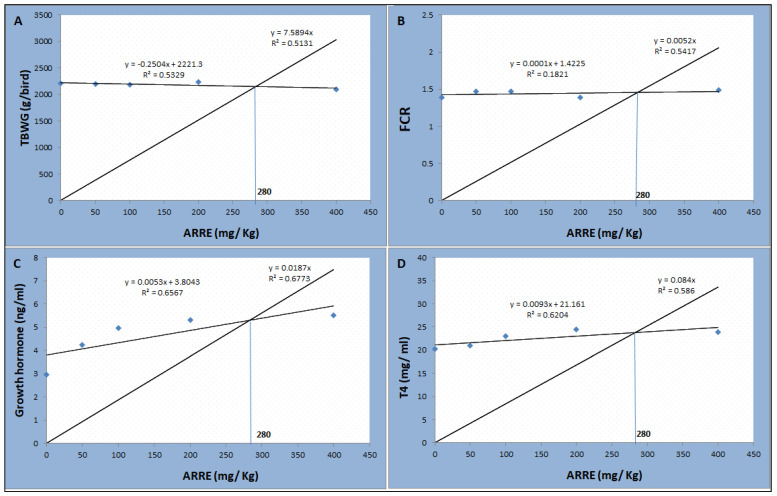
The broken-line regression model shows the optimum dietary level of ARRE addition based on the data of TBWG (**A**), FCR (**B**), growth hormone (**C**), and T4 (**D**).

As shown in Table 7, the serum T4 level was linearly higher in the ARRE200 group (*p* < 0.01). The serum level of growth hormone was linearly and quadratically increased in a level-dependent manner, where the highest levels were observed in ARRE200 and ARRE400 groups (*p* < 0.05). The serum levels of glucose, leptin, AST, ALT, creatinine, and urea were not significantly different between all groups (*p* > 0.05). According to the broken-line regression analysis, the optimum inclusion level of ARRE was 280 mg Kg^−1^ based on the data of total body weight gain, feed conversion ratio, growth hormone, and T4 levels (Figure 4).

### 3.9. Lipid Profile

All levels of supplemental ARRE linearly and quadratically decreased the serum triglyceride level compared with the control group (*p* < 0.05). While the TC, HDL-C, LDL-C, and VLDL-C levels were not significantly different between all groups (*p* > 0.05) (Table 8).

### 3.10. Antioxidant Capacity

The serum TAC level was linearly and quadratically increased significantly in the ARRE100-ARRE400 groups compared with the control group (*p* < 0.01). The serum activity of CAT and SOD were linearly raised in the ARRE400 and ARRE200 groups, respectively, compared with the control group (*p* < 0.05). The serum MDA level was linearly decreased by increasing the level of ARRE (*p* < 0.01) (Table 9).

### 3.11. Immune Status

As shown in Table 10, the total protein level in the serum level was linearly increased in ARRE200 compared with the control group (*p* < 0.01). At the same time, the serum albumin and globulin levels were not significantly different between groups (*p* > 0.05). Supplemental ARRE linearly and quadratically increased the serum levels of lysozymes and IL10 compared with the control group (*p* < 0.01). The serum C3 level was linearly increased in ARRE200 and ARRE400 groups (*p* = 0.01).

## 4. Discussion

### 4.1. Effect of ARRE on the Growth Performance

Studying the effects of feeding systems on birds’ performance, health, and gut histomorphology is essential for developing approaches that use antibiotic alternatives in poultry manufacture. The current study investigated the effects of using anthocyanin-rich roselle extract additive on the growth, carcass traits, meat composition, intestinal morphology, blood biochemical parameters, antioxidant activity, and immune status of broiler chickens. In the present study, *Hibiscus* acidified ethanolic extract was recorded with total anthocyanin content (TAC) of about 359.3 mg cyanidin 3-glucoside/100 g dw, total polyphenol concentration (TPC) of about 598 mg gallic acid equivalent (GAE)/100 g dw, and total flavonoids (TFs) of about 100 mg quercetin equivalent (QE)/100 g dw.

Dietary supplementation with polyphenols did not exert a specific effect on the animal’s growth, which was increased, decreased, or unaffected, relying on the feed’s included compound [63]. Although dietary ARRE improved most intestinal morphometric measures, the growth hormone, and thyroxin hormone, its addition did not affect the birds’ growth performance or carcass traits, except for decreased feed intake and improved FCR during the starter period, and decreased BW and BWG during the grower period in the ARRE400 group. This may be due to the minor effect of ARRE on nutrient digestion. The optimum inclusion level of ARRE was 280 mg Kg^−1^ based on the data of TBWG, FCR, growth hormone, and T4 levels. It has been reported that polyphenols can cause inhibition of digestive enzyme secretion, increased protein secretion, and decreased protein and amino acids digestibility, leading to opposing metabolic effects demonstrated by reduced body weight and feeding efficiency [64,65]. Dietary ARRE reduces pancreatic lipase activity and reduces fat digestion and absorption in the intestine [66]. Earlier studies indicated that polyphenols inhibit various enzymes involving α-amylase and α-glucosidase activity [67,68] pancreatic lipase [69]. The inhibition of digestive enzymes may be due to insoluble complexes from the polymeric polyphenols and the proteins in the gastrointestinal tract [70]. Csernus et al. [30] conveyed no effect of anthocyanin on the body weight and average daily gain of broilers, only it increased the average daily feed intake during the grower period. Nasrawi [71] reported improved growth of broiler chickens by roselle flower powder. He attributed these results to the active compounds in roselle, such as anthocyanin and vitamin C, which significantly stimulate the thyroid gland and growth hormone secretion and improve metabolism [72].

### 4.2. Chemical and Fatty Acid Composition of the Breast Muscles

There is little research on the effect of ARRE on the chemical and fatty acid composition in the breast muscles of chickens. The present study demonstrated that dietary ARRE did not affect the chemical composition of breast muscle, which is consistent with the growth performance results. Dietary ARRE increased the ω-3 PUFA percentage and ω-3 to the ω-6 ratio level dependency. Prommachart et al. [73] reported higher n-3 and n-6 PUFA in the meat of beef cattle-fed anthocyanin-rich extract from black rice and purple corn, which denotes healthier meat for the consumer. Villasante et al. [74] found an increase in the percentages of total n-3 PUFA in the plasma of rainbow trout-fed anthocyanins-rich purple corn extract. Jaturasitha et al. [75] reported a high rate of n-3 PUFA in raw loin chops of pigs fed anthocyanins-rich purple rice extract, indicating bioactive compounds such as anthocyanins in ARRE may affect the meat content from n-3 and n-6 PUFA. Anthocyanins may encourage the conversion of α-linolenic acid to EPA and DHA [73]. Changes in the fatty acid profile occur by reducing the saturated fatty acids concentrations and increasing PUFAs levels in the meat, promoting consumer health by increasing the food quality and oxidative stability [76].

### 4.3. Effect of ARRE on the Intestinal Morphology

Intestinal morphometric measures can denote digestive functions. Increased VH and decreased CD can support more extensive nutrient digestion and absorption [4,77]. Dietary ARRE increased the villus height and goblet cell count, and the ARRE100 group increased the villus height to crypt depth ratio, indicating a positive effect in absorption functions. Csernus et al. [30] reported increased ileal VH, VH: CD ration, and mucosal thickness by anthocyanin supplementation in broiler diets. Polyphenols have low bioavailability, and unabsorbed compounds significantly impact gut health [78,79]. Polyphenols boost the host’s immune system and overall health by promoting the growth of beneficial bacteria (*Lactobacillus* spp., *Bacillus* spp.) and stabilization of the intestinal microflora [80]. It can have an optimistic effect on gut morphology and enhance nutrient absorption in monogastric animals [16].

### 4.4. Effect of ARRE on the Blood Hematology

The data of the blood hematology presented increased RBCs count in the ARRE100 group, decreased platelets count in the ARRE100 and ARRE 200 groups, decreased MCV value in the ARRE100 group, and decreased MCH value in the ARRE50 group. The heterophils and eosinophils counts were higher in the ARRE400 and ARRE50 groups, respectively. However, the reported values are within the normal range of healthy birds [81,82]. Other hematological indices (HB, PCV, WBCs, lymphocytes, basophils, and monocytes) were not changed by dietary ARRE, indicating sufficient nutrient release for electrophoresis and the blood’s ability to carry and release oxygen was not adversely affected [83]. Similarly, Ugwu et al. [84] detected no significant impact of *H. sabdariffa* calyx extract supplementation on the blood hematology of the birds, except increased MCV value than the normal range. Asaniyan and Akinduro [85] also reported a nonsignificant effect of aqueous extract of roselle plant on the HB, platelets count, PCV, and RBCs count of broiler chickens.

### 4.5. Effect of ARRE on the Clinic-Biochemical Indices

Assessment of blood biochemistry in poultry gives valuable data about their nutritional, performance, and health status [86]. The current study showed higher T4 levels in the ARRE200 group and higher growth hormone levels by ARRE addition in a level-dependent manner, where the highest levels were observed in the ARRE200 and ARRE400 groups. These results were attributed to the bioactive compounds in the roselle extract (anthocyanin) that have a significant role in stimulating the thyroid gland and improving metabolism [72]. Our results showed no effect of dietary ARRE addition on glucose and leptin hormone levels. The constancy in the serum glucose indicates that dietary ARRE did not affect glucose intake and consequent metabolism by the cells. Dietary ARRE did not alter the serum AST and ALT. The constancy in the liver enzymes indicates that it did not alter the liver function and integrity because these enzymes (AST and ALT) are hepatotoxicity biomarkers [81]. The ARRE200 group increased the serum total protein level compared with other groups with no effect of supplemental ARRE on serum albumin and globulin. Total serum protein and albumin are considered a measure of bioproduction of plasma proteins by the liver [85].

Dietary ARRE did not change the serum urea and creatinine. Creatinine is a relatively dependable indicator of kidney function. The constancy in the serum urea and creatinine levels indicates that dietary ARRE did not adversely impact the kidney of the animals. Similarly, Ugwu et al. [84] observed no significant effect of *H. sabdariffa* calyx extract on broiler chickens’ glucose, AST, ALT, urea, and creatinine levels. All levels of supplemental ARRE decreased the serum triglyceride level with no effect on the TC level. This may be due to the inhibition of the pancreatic lipase activity by anthocyanin and so reduction of lipid digestion and absorption [69]. Chen et al. [66] reported decreased TG level in broiler chicken by dietary *Hibiscus sabdariffa* extract.

### 4.6. Effect of ARRE on the Antioxidant Activity

The current study revealed an increase in the serum TAC in the ARRE100-ARRE400 groups, increased SOD activity in the ARRE200 group, and decreased MDA level by increasing the level of ARRE. Anthocyanins have a high thermostability and influence antioxidative, anti-inflammatory, hepatoprotective, and cardioprotective activities [87]. The antioxidant effect may be due to the role of the phenolic compounds in scavenging the free radicals by forming complexes with metal ions and inhibiting the singlet oxygen formation [88,89]. Polyphenols’ antioxidant activities can also be initiated by having hydrogen from hydroxyl groups located along the aromatic ring to cease the free-radical oxidation of biomolecules. Polyphenols also inhibit oxidases, decrease α-tocopherol radicals, and activate antioxidant enzymes [90]. Our results are aligned with Wang et al. [91], who informed increased antioxidant enzymes activity involving total superoxide dismutase (T-SOD) and glutathione peroxidase (GSH-Px), and reduced MDA in the tissues and blood of chickens by proanthocyanidins extracted from bilberry plant, thus reducing the lipid oxidation and oxidative damage.

### 4.7. Impact of ARRE on the Immune Response

The immune response can be assessed in several ways, for example, quantification of leukocyte pools, antibody concentrations, cytokine responses, inflammatory markers, phagocytic abilities, and masses of different immune organs. Several approaches have been used to improve chickens’ immune and inflammatory responses [1]. In the present study, the relative weights of the immune organs (spleen and bursa of Fabricius) were not affected by ARRE supplementation. In addition, Csernus et al. [30] reported no effect of dietary anthocyanin on the lymphoid organ’s weight (spleen). Park et al. [92] noticed increased immune cells in the thymus, spleen, and bursa of Fabricius, decreased T cytokines expression, and boosted immune function in chickens by proanthocyanidins extracted from pine bark.

Chicken IgY, or chicken IgG, is a significant immunoglobulin that can counteract viruses, bacteria, or toxins with complement activation [93]. In the present study, dietary ARRE raised the immunoexpression of IgG in the spleen, indicating enhanced humoral immunity. Cytokines act as extracellular signals between cells during the immune responses [94]. Supplemental ARRE significantly increased the serum levels of lysozymes and IL10. The serum C3 level was increased in ARRE200 and ARRE400 groups. Increasing these immune indicators indicate the immune-modulating effect of ARRE. This enhancement was attributed to the flavonoids content of the extract, mainly anthocyanin, as it has been reported that flavonoids enhance the humoral immune response set by the chickens [95].

## 5. Conclusions

Dietary anthocyanin-rich roselle extracts did not affect the bird’s growth and carcass traits. However, its addition had many beneficial effects, such as improving the bird’s intestinal morphology indicated by increased villous height and VH: CD ratio, indicating better nutrient absorption. Moreover, it improved the birds’ metabolic functions indicated by increased T4 and growth hormone with no effect on the glucose and leptin hormone. Dietary ARRE lowered the triglyceride level and improved the antioxidant activity by increasing the TAC and SOD activity and reducing the MDA level. ARRE addition improved the immune status by increasing the serum levels of IL10 and lysozyme and upregulating the IgG immune expression in the spleen. Furthermore, its addition increased the n-3 PUFA content in the breast muscles, increasing consumer acceptance. The optimum inclusion level of ARRE was 280 mg Kg^−1^.

## Figures and Tables

**Table 1:** The proximate chemical composition of the basal diet as fed basis (%).

Ingredients	Unit	Starter	Grower	Finisher
Corn 7.5% cp	%	55.8	59.29	62.26
Soybean meal 47% cp	%	33.7	28.085	23.735
Corn gluten meal 60% cp	%	3.75	5.3	5.9
Oil (soya)—e76	%	2.2	3.0	4.0
Dicalcium phosphate dcp 18%	%	1.5	1.4	1.3
Calcium carbonate	%	1.2	1.2	1.1
Sodium bicarbonate	%	0.25	0.25	0.25
Dl methionine 99%	%	0.4	0.3	0.33
Broiler premix *	%	0.3	0.3	0.3
L-LYSINE HCL 98%	%	0.47	0.45	0.4
Salt	%	0.15	0.15	0.15
Antimycotoxin	%	0.1	0.1	0.1
Choline 60 veg	%	0.07	0.07	0.07
L-THREONINE 98.5%	%	0.1	0.1	0.1
Enzyme Phytase	%	0.005	0.005	0.005
Chemical analysis **				
Moisture	%	11.25	11.17	11.23
ME poultry. (kcal/kg)	Kcal/kg	3000.03	3100.64	3200.78
Crude protein	%	23.06	21.53	20.02
Lysine	%	1.47	1.31	1.15
Methionine	%	0.72	0.61	0.62
Calcium	%	0.94	0.90	0.83
Av. Phosphorus	%	0.48	0.44	0.41

* Premix per kg of diet: vitamin A, 1500 IU; vitamin D3, 200 IU; vitamin E, 10 mg; vitamin K3, 0.5 mg; thiamine, 1.8 mg; riboflavin, 3.6 mg; ** pantothenic acid, 10 mg; folicacid, 0.55 mg; pyridoxine, 3.5 mg; niacin, 35 mg; cobalamin, 0.01 mg; biotin, 0.15 mg; Fe, 80 mg; Cu, 8 mg; Mn, 60 mg; Zn, 40 mg; I, 0.35 mg; Se, 0.15 mg.

**Table 2:** The effect of dietary addition of ARRE on the growth of broiler chickens.

Parameters	ARRE0	ARRE50	ARRE100	ARRE200	ARRE400	SEM	Regression #	
							Linear	Quadratic
IBW (g)	89.04	87.08	86.66	88.12	88.33	0.32	0.06	0.33
Starter period								
BW (g)	314.17	310.28	305.83	316.94	305.28	2.91	0.93	0.62
BWG (g)	225.12	223.19	219.17	228.82	216.94	2.80	0.94	0.46
FI (g)	250.48 ^a^	243.54 ^a,b^	240.83 ^a,b^	245.21 ^a,b^	228.54 ^b^	2.88	0.03	0.56
FCR	1.11 ^a^	1.09 ^a,b^	1.10 ^a,b^	1.07 ^a,b^	1.05 ^b^	0.009	0.01	0.58
Grower period								
BW (g)	1170.17 ^a^	1138.61 ^a^	1086.39 ^a,b^	1141.67 ^a,b^	1063.89 ^b^	13.65	0.01	0.23
BWG (g)	856.00 ^a^	828.33 ^a^	780.56 ^a,b^	824.72 ^ab^	758.61 ^b^	12.62	0.01	0.28
FI (g)	1102.14	1098.54	1094.05	1046.88	1063.13	12.74	0.20	0.97
FCR	1.29	1.33	1.40	1.27	1.40	0.019	0.08	0.22
Finisher period								
BW (g)	2293.72	2288.61	2270.28	2324.17	2181.11	35.09	0.87	0.80
BWG (g)	1123.56	1150.00	1183.89	1182.50	1117.22	31.11	0.27	0.86
FI (g)	1707.62	1883.75	1870.95	1820.00	1818.45	33.08	0.52	0.19
FCR	0.74	0.82	0.82	0.78	0.83	0.013	0.39	0.09
Overall performance								
BW (g)	2293.72	2288.61	2270.28	2324.17	2181.11	25.68	0.87	0.80
BWG (g)	2204.67	2201.53	2183.61	2236.04	2092.78	35.09	0.87	0.78
FI (g)	3060.24	3225.83	3205.83	3112.08	3110.12	37.62	0.96	0.24
FCR	1.39	1.47	1.47	1.39	1.49	0.017	0.69	0.18
PER	3.46	3.29	3.28	3.46	3.24	0.03	0.63	0.19
RGR	185.02	185.33	185.21	185.33	184.40	0.23	0.86	0.44

# The regressions were considered significant at *p* < 0.05. Variation in the data was expressed as pooled SEM. ^a,b^ means within the same row carrying different superscripts are significantly different (*p* < 0.05). IBW—initial body weight, BW—body weight, BWG—body weight gain, FI—feed intake, FCR—feed conversion ratio, PER—protein efficiency ratio, RGR—relative growth rate.

**Table 3:** The effect of dietary supplementation of ARRE on the carcass traits (%) relative to live weight.

Parameters	ARRE0	ARRE50	ARRE100	ARRE200	ARRE400	SEM	Regression #	
							Linear	Quadratic
Carcass	64.29	64.56	68.71	65.35	63.41	0.64	0.84	0.05
Intestine	5.51	4.67	5.58	5.10	5.73	0.19	0.54	0.35
Spleen	0.09	0.11	0.12	0.09	0.11	0.006	0.74	0.83
Bursa	0.161	0.162	0.163	0.148	0.132	0.008	0.27	0.50
Gizzard	1.41	2.20	1.94	2.16	2.46	0.14	0.05	0.66
Liver	2.01	1.94	1.75	1.89	2.16	0.06	0.55	0.07

# The regressions were considered significant at *p* < 0.05. Variation in the data was expressed as pooled SEM.

**Table 4:** The effect of dietary supplementation of ARRE on the chemical and fatty acid composition of the breast muscle.

Parameters	ARRE0	ARRE50	ARRE100	ARRE200	ARRE400	SEM	Regression #	
							Linear	Quadratic
Dry matter%	69.59	72.31	74.68	74.42	73.19	0.43	0.18	0.27
Crude protein%	67.27	67.25	67.1	66.7	67.85	1.30	0.65	0.68
Fat%	5.92	4.9	6.0	5.8	5.45	0.20	0.96	0.94
Ash%	4.1	4.5	3.66	5.2	4.15	0.48	0.36	0.93
ω-3 (%)	0.09 ^c^	0.11b^c^	0.15 ^a^^,^^b,c^	0.16 ^a,b^	0.19 ^a^	0.01	<0.01	0.94
ω-6 (%)	2.00	2.05	2.07	2.06	2.09	0.01	0.09	0.56
ω-3: ω-6 ratio	0.04^b^	0.05^b^	0.07 ^a,b^	0.07 ^a,b^	0.09 ^a^	0.004	<0.01	0.92

# The regressions were considered significant at *p* < 0.05. Variation in the data was expressed as pooled SEM. ^a,b,c^ means within the same row carrying different superscripts are significantly different (*p* < 0.05). ω-3 (% of total fatty acids): omega-3 fatty acids. ω-6 (% of total fatty acids): omega-6 fatty acids.

**Table 5:** The effect of dietary supplementation of ARRE on the morphometric measures (µm) of the small intestine of broiler chickens.

Parameters	ARRE0	ARRE50	ARRE100	ARRE200	ARRE400	SEM	Regression #	
							Linear	Quadratic
Duodenum								
VH	331.12 ^b^	551.84 ^a^	605.48 ^a^	489.09 ^a,b^	485.71 ^a,b^	28.03	0.05	<0.01
VW	87.74	94.13	119.79	78.29	108.81	5.86	0.48	0.66
CD	79.91	110.93	85.21	108.45	102.28	4.52	0.12	0.41
VH:CD	4.12 ^b^	4.97 ^b^	7.18 ^a^	4.49 ^b^	4.86 ^b^	0.32	0.45	<0.01
GCC	104.60 ^b^	137.02 ^a,b^	150.83 ^a^	124.24 ^a,b^	121.48 ^a,b^	4.98	0.38	<0.01
Jejunum								
VH	882.72 ^b^	1079.67 ^a,b^	1139.82 ^a^	946.82 ^a,b^	921.59 ^a,b^	32.61	0.74	<0.01
VW	119.42	116.38	110.27	102.84	107.60	5.40	0.41	0.78
CD	150.82	145.76	125.17	108.85	135.70	5.37	0.60	0.35
VH:CD	7.06	7.40	7.54	9.28	6.80	0.39	0.62	0.23
GCC	201.53 ^c^	246.60 ^b^	309.44 ^a^	234.98 ^b^	228.10 ^b,c^	9.82	0.04	<0.01
Ileum								
VH	487.12 ^c^	723.85 ^a,b^	752.83 ^a^	600.64 ^a,b,c^	542.24 ^b,c^	31.21	0.92	<0.01
VW	161.46 ^a^	138.20 ^a,b^	110.48 ^a,b^	138.89 ^a,b^	101.04 ^b^	7.38	0.01	0.56
CD	74.97 ^b^	108.78 ^a^	114.03 ^a^	97.47 ^a,b^	102.69 ^a,b^	4.32	0.05	<0.01
VH:CD	6.77	6.63	6.60	6.14	5.31	0.24	0.06	0.37
GCC	64.63 ^c^	96.94 ^b^	148.25 ^a^	113.13 ^b^	103.48 ^b^	7.42	<0.01	<0.01

# The regressions were considered significant at *p* < 0.05. Variation in the data was expressed as pooled SEM. ^a,b,c^ means within the same row carrying different superscripts are significantly different at (*p <* 0.05). VH—villous height, VW—villous width, CD—crypt depth, VH:CD—villous height to crypt depth ratio, GCC—goblet cell count.

**Table 6:** The effect of dietary supplementation of ARRE on the blood hematology of broiler chickens.

Parameters	ARRE0	ARRE50	ARRE100	ARRE200	ARRE400	SEM	Regression #	
							Linear	Quadratic
RBCs (×10^6^/µL)	2.79 ^b^	3.35 ^a,b^	3.91 ^a^	3.20 ^a,b^	2.84 ^b^	0.15	0.92	<0.01
Hb (g/dL)	8.75	8.90	10.63	9.77	9.25	0.29	0.33	0.10
PCV%	33.70	34.54	36.41	35.66	34.78	0.37	0.20	0.06
Platelets (×10^3^/µL)	22.33 ^a^	18.67 ^a,b^	14.33 ^b,c^	10.67 ^c^	19.33 ^a,b^	1.24	0.01	<0.01
MCV (fL)	122.33 ^a^	103.04 ^a,b^	93.46 ^b^	112.16 ^a,b^	122.77 ^a^	3.65	0.58	<0.01
MCH (Pg)	31.62 ^a,b^	26.54 ^c^	27.03 ^b,c^	30.63 ^a,b,c^	32.61 ^a^	0.76	0.08	<0.01
MCHC (g/dL)	23.94	25.76	29.05	27.38	26.59	0.68	0.14	0.07
WBCs (×10^3^/µL)	18.87	20.00	18.67	19.66	18.67	0.22	0.61	0.27
Lymphocytes (×10^3^/µL)	10.54	10.87	10.78	11.16	10.31	0.15	0.83	0.08
Heterophils (×10^3^/µL)	5.90 ^a,b^	6.69 ^a,b^	5.76 ^b^	6.18 ^a,b^	6.84 ^a^	0.15	0.06	0.18
Monocytes (×10^3^/µL)	1.60	1.53	1.46	1.55	1.39	0.13	0.10	0.98
Eosinophils (×10^3^/µL)	0.52 ^a,b^	0.58 ^a^	0.41 ^a,b^	0.49 ^a,b^	0.39 ^b^	0.02	0.02	0.65
Basophils (×10^3^/µL)	0.31	0.32	0.26	0.29	0.27	0.01	0.33	0.79

# The regressions were considered significant at *p* < 0.05. Variation in the data was expressed as pooled SEM. ^a,b,c^ means within the same row carrying different superscripts are significantly different (*p* < 0.05). RBCs—Red blood cells; Hb—hemoglobin; PCV—packed cell volume; MCV—mean corpuscular volume; MCH—mean corpuscular hemoglobin; MCHC—mean corpuscular hemoglobin concentration; WBCs—White blood cells.

**Table 7:** The effect of dietary supplementation of ARRE on the blood biochemical parameters of broiler chickens.

Parameters	ARRE0	ARRE50	ARRE100	ARRE200	ARRE400	SEM	Regression #	
							Linear	Quadratic
T3 (ng/mL)	3.71	3.88	4.15	4.09	4.33	0.10	0.06	0.82
T4 (ng/mL)	20.22 ^b^	21.00 ^a,b^	23.09 ^a,b^	24.50 ^a^	23.97 ^a,b^	0.56	<0.01	0.35
Glucose (mg/dL)	336.67	338.67	341.67	339.33	339.33	1.56	0.64	0.54
GH (ng/mL)	2.97 ^c^	4.23 ^b^	4.97 ^a,b^	5.30 ^a^	5.53 ^a^	0.26	<0.01	0.01
Leptin (ng/mL)	2.18	1.65	1.63	1.97	2.01	0.10	0.98	0.11
ALT (U/L)	6.00	6.67	6.67	6.33	8.00	0.39	0.24	0.64
AST (U/L)	49.00	49.00	57.00	51.67	53.00	1.97	0.49	0.56
Creatinine (mg/dL)	0.23	0.25	0.24	0.25	0.24	0.003	0.06	0.12
Uric acid (mg/dL)	3.13	3.53	3.40	3.13	3.33	0.09	0.98	0.53

# The regressions were considered significant at *p* < 0.05. Variation in the data was expressed as pooled SEM. ^a,b,c^ Means within the same row carrying different superscripts are significantly different at (*p* < 0.05). Triiodothyronine (T3); Thyroxine (T4); Growth hormone (GH); Aspartate aminotransferase (AST); Alanine aminotransferase (ALT).

**Table 8:** The effect of dietary supplementation of ARRE on the serum lipid profile of broiler chickens.

Parameters	ARRE0	ARRE50	ARRE100	ARRE200	ARRE400	SEM	Regression #	
							Linear	Quadratic
TC (mmol/L)	3.54	3.33	3.41	3.42	3.40	0.02	0.29	0.18
HDL-C (mmol/L)	1.99	2.17	2.11	2.04	2.11	0.02	0.45	0.22
LDL-C (mmol/L)	1.31	0.93	1.08	1.15	1.07	0.04	0.34	0.14
VLDL-C (mmol/L)	0.24	0.23	0.22	0.23	0.22	0.003	0.14	0.44
TG (mmol/L)	1.26 ^a^	1.16 ^b^	1.16 ^b^	1.17 ^b^	1.15 ^b^	0.01	<0.01	0.03

# The regressions were considered significant at *p* < 0.05. Variation in the data was expressed as pooled SEM. ^a,b^ means within the same row carrying different superscripts are significantly different (*p* < 0.05). TC—Total cholesterol; TG—triglycerides; HDL-C—high-density lipoprotein cholesterol; LDL-C—low-density lipoprotein cholesterol; VLDL-C—very low-density lipoprotein cholesterol.

**Table 9:** The effect of dietary supplementation of ARRE on the serum antioxidant activity of broiler chickens.

Parameters	ARRE0	ARRE50	ARRE100	ARRE200	ARRE400	SEM	Regression #	
							Linear	Quadratic
TAC (U/mL)	10.76 ^b^	10.66 ^b^	13.25 ^a^	13.26 ^a^	13.09 ^a^	0.34	<0.01	0.02
CAT (U/mL)	4.42 ^b^	4.54 ^a,b^	5.74 ^a,b^	5.92 ^a,b^	6.45 ^a^	0.30	0.01	0.93
SOD (U/mL)	140.09 ^b^	145.24 ^a,b^	155.29 ^a,b^	158.71 ^a^	155.27 ^a,b^	2.36	<0.01	0.12
MDA (nmol/mL)	6.67 ^a^	5.80 ^a,b^	3.60 ^b,c^	3.27 ^c^	3.23 ^c^	0.43	<0.01	0.07

# The regressions were considered significant at *p* < 0.05. Variation in the data was expressed as pooled SEM. ^a,b,c^ means within the same row carrying different superscripts are significantly different (*p* < 0.05). TAC—total antioxidant capacity; CAT—catalase; SOD—superoxide dismutase; MDA—Malondialdehyde.

**Table 10:** The effect of dietary supplementation of ARRE on the immune indices of broiler chickens.

Parameters	ARRE0	ARRE50	ARRE100	ARRE200	ARRE400	SEM	Regression #	
								Linear	Quadratic
TP (g/dL)	3.73 ^b^	4.02 ^a,b^	4.51 ^a,b^	4.90 ^a^	4.83 ^a,b^	0.15	<0.01	0.39
ALB (g/dL)	1.28	1.31	1.90	1.75	1.66	0.11	0.13	0.27
GLU (g/dL)	2.45	2.71	2.61	3.15	3.17	0.13	0.06	0.88
Lysozyme (µg/mL)	133.00 ^b^	173.00 ^a^	181.67 ^a^	184.67 ^a^	189.67 ^a^	5.67	<0.01	<0.01
IL10 (pg/mL)	1.60 ^b^	3.63 ^a^	4.03 ^a^	3.73 ^a^	4.30 ^a^	0.26	<0.01	<0.01
C3 (mg/dL)	1.11 ^b^	1.20 ^a,b^	1.22 ^a,b^	1.23 ^a^	1.24 ^a^	0.01	0.01	0.09

# The regressions were considered significant at *p* < 0.05. Variation in the data was expressed as pooled SEM. ^a,b^ means within the same row carrying different superscripts are significantly different (*p* < 0.05), TP—total protein; ALB—albumin; GLU—globulin, C3—complement 3; IL10—interleukin 10.

## Data Availability

Data is contained in the manuscript.

## References

[1] Hasted T.-L., Sharif S., Boerlin P., Diarra M.S. (2021). Immunostimulatory Potential of Fruits and Their Extracts in Poultry. Front. Immunol..

[2] Amer S.A., Mohamed W.A., Gharib H.S., Al-Gabri N.A., Gouda A., Elabbasy M.T., Abd El-Rahman G.I., Omar A.E. (2021). Changes in the growth, ileal digestibility, intestinal histology, behavior, fatty acid composition of the breast muscles, and blood biochemical parameters of broiler chickens by dietary inclusion of safflower oil and vitamin C. BMC Vet. Res..

[3] Amer S.A., A-Nasser A., Al-Khalaifah H., AlSadek D., Fattah D., Roushdy E., Sherief W., Farag M., Altohamy D., Abdel-Wareth A. (2021). Effect of Dietary Medium-Chain α-Monoglycerides on the Growth Performance, Intestinal Histomorphology, Amino Acid Digestibility, and Broiler Chickens’ Blood Biochemical Parameters. Animals.

[4] Amer S.A., Beheiry R.R., Abdel Fattah D.M., Roushdy E.M., Hassan F.A., Ismail T.A., Zaitoun N., Abo-Elmaaty A., Metwally A.E. (2021). Effects of different feeding regimens with protease supplementation on growth, amino acid digestibility, economic efficiency, blood biochemical parameters, and intestinal histology in broiler chickens. BMC Vet. Res..

[5] Amer S.A., Naser M.A., Abdel-Wareth A.A., Saleh A.A., Elsayed S.A., Abdel Fattah D.M., Metwally A.E. (2020). Effect of dietary supplementation of alpha-galactosidase on the growth performance, ileal digestibility, intestinal morphology, and biochemical parameters in broiler chickens. BMC Vet. Res..

[6] Zeng Z., Zhang S., Wang H., Piao X. (2015). Essential oil and aromatic plants as feed additives in non-ruminant nutrition: A review. J. Anim. Sci. Biotechnol..

[7] Omar A.E., Al-Khalaifah H.S., Mohamed W.A., Gharib H.S., Osman A., Al-Gabri N.A., Amer S.A. (2020). Effects of phenolic-rich onion (*Allium cepa* L.) extract on the growth performance, behavior, intestinal histology, amino acid digestibility, antioxidant activity, and the immune status of broiler chickens. Front. Vet. Sci..

[8] Al-Khalaifah H., Khalil A.A., Amer S.A., Shalaby S.I., Badr H.A., Farag M.F., Altohamy D.E., Abdel Rahman A.N. (2020). Effects of Dietary Doum Palm Fruit Powder on Growth, Antioxidant Capacity, Immune Response, and Disease Resistance of African Catfish, Clarias gariepinus (B.). Animals.

[9] Da-Costa-Rocha I., Bonnlaender B., Sievers H., Pischel I., Heinrich M. (2014). Hibiscus sabdariffa L.–A phytochemical and pharmacological review. Food Chem..

[10] Müller B., Kraus J., Franz G. (1989). Polysaccharides from Hibiscus sabdariffa: Structural Investigation and Biological Activity. Planta Med..

[11] Eggensperger H., Wilker M. (1996). Hibiscus-Extrakt: Ein hautverträglicher Wirkstoffkomplex aus AHA’s und polysacchariden. Teil 1. Parfümerie Kosmet..

[12] Khoo H.E., Azlan A., Tang S.T., Lim S.M. (2017). Anthocyanidins and anthocyanins: Colored pigments as food, pharmaceutical ingredients, and the potential health benefits. Food Nutr. Res..

[13] Varga M., Bánhidy J., Cseuz L., Matuz J. (2013). The anthocyanin content of blue and purple coloured wheat cultivars and their hybrid generations. Cereal Res. Commun..

[14] Mrkvicová E., Pavlata L., Karásek F., Šťastník O., Doležalová E., Trojan V., Vyhnánek T., Hřivna L., Holeksová V., Mareš J. (2017). The influence of feeding purple wheat with higher content of anthocyanins on antioxidant status and selected enzyme activity of animals. Acta Vet. Brno.

[15] Prior R.L., Wu X., Gu L., Hager T.J., Hager A., Howard L.R. (2008). Whole berries versus berry anthocyanins: Interactions with dietary fat levels in the C57BL/6J mouse model of obesity. J. Agric. Food Chem..

[16] Kamboh A., Arain M.A., Mughal M.J., Zaman A., Arain Z., Soomro A. (2015). Flavonoids: Health promoting phytochemicals for animal production-a review. J. Anim. Health Prod..

[17] Kowalczyk E., Kopff A., Fijałkowski P., Kopff M., Niedworok J., Błaszczyk J., Kêdziora J., Tyślerowicz P. (2003). Effect of anthocyanins on selected biochemical parameters in rats exposed to cadmium. Acta Biochim. Pol..

[18] Kim A.-J., Park S. (2006). Mulberry extract supplements ameliorate the inflammation-related hematological parameters in carrageenan-induced arthritic rats. J. Med. Food.

[19] Xia X., Ling W., Ma J., Xia M., Hou M., Wang Q., Zhu H., Tang Z. (2006). An anthocyanin-rich extract from black rice enhances atherosclerotic plaque stabilization in apolipoprotein E–deficient mice. J. Nutr..

[20] Yilmaz E. (2019). Effects of dietary anthocyanin on innate immune parameters, gene expression responses, and ammonia resistance of Nile tilapia (Oreochromis niloticus). Fish Shellfish Immunol..

[21] Boto-Ordóñez M., Urpi-Sarda M., Queipo-Ortuño M.I., Tulipani S., Tinahones F.J., Andres-Lacueva C. (2014). High levels of Bifidobacteria are associated with increased levels of anthocyanin microbial metabolites: A randomized clinical trial. Food Funct..

[22] Ma Y., Ding S., Fei Y., Liu G., Jang H., Fang J. (2019). Antimicrobial activity of anthocyanins and catechins against foodborne pathogens Escherichia coli and Salmonella. Food Control.

[23] Anhê F.F., Roy D., Pilon G., Dudonné S., Matamoros S., Varin T.V., Garofalo C., Moine Q., Desjardins Y., Levy E. (2015). A polyphenol-rich cranberry extract protects from diet-induced obesity, insulin resistance and intestinal inflammation in association with increased Akkermansia spp. population in the gut microbiota of mice. Gut.

[24] Timberlake C., Bridle P. (1982). Distribution of Anthocyanins in Food Plants.

[25] Mannino G., Perrone A., Campobenedetto C., Schittone A., Bertea C.M., Gentile C. (2020). Phytochemical profile and antioxidative properties of Plinia trunciflora fruits: A new source of nutraceuticals. Food Chem..

[26] Mazza G., Miniati E. (2018). Anthocyanins in Fruits, Vegetables, and Grains.

[27] Mannino G., Gentile C., Ertani A., Serio G., Bertea C.M. (2021). Anthocyanins: Biosynthesis, distribution, ecological role, and use of biostimulants to increase their content in plant foods—A review. Agriculture.

[28] Mannino G., Gentile C., Maffei M.E. (2019). Chemical partitioning and DNA fingerprinting of some pistachio (*Pistacia vera* L.) varieties of different geographical origin. Phytochemistry.

[29] Changxing L., Chenling M., Alagawany M., Jianhua L., Dongfang D., Gaichao W., Wenyin Z., Syed S., Arain M., Saeed M. (2018). Health benefits and potential applications of anthocyanins in poultry feed industry. World’s Poult. Sci. J..

[30] Csernus B., Biró S., Babinszky L., Komlósi I., Jávor A., Stündl L., Remenyik J., Bai P., Oláh J., Pesti-Asbóth G. (2020). Effect of carotenoids, oligosaccharides and anthocyanins on growth performance, immunological parameters and intestinal morphology in broiler chickens challenged with Escherichia coli lipopolysaccharide. Animals.

[31] Zhao X., Corrales M., Zhang C., Hu X., Ma Y., Tauscher B. (2008). Composition and thermal stability of anthocyanins from Chinese purple corn (*Zea mays* L.). J. Agric. Food Chem..

[32] Vulić J.J., Tumbas V.T., Savatović S.M., Đilas S.M., Ćetković G.S., Čanadanović-Brunet J.M. (2011). Polyphenolic content and antioxidant activity of the four berry fruits pomace extracts. Acta Period. Technol..

[33] Wu H.-Y., Yang K.-M., Chiang P.-Y. (2018). Roselle anthocyanins: Antioxidant properties and stability to heat and pH. Molecules.

[34] Singleton V.L., Orthofer R., Lamuela-Raventós R.M. (1999). Analysis of total phenols and other oxidation substrates and antioxidants by means of folin-ciocalteu reagent. Methods Enzymol..

[35] Ordonez A., Gomez J., Vattuone M. (2006). Antioxidant activities of Sechium edule (Jacq.) Swartz extracts. Food Chem..

[36] Durst R.W., Wrolstad R.E. (2001). Separation and characterization of anthocyanins by HPLC. Curr. Protoc. Food Anal. Chem..

[37] AVIAGEN R. (2009). Ross Broiler Management Manual. http://pt.aviagen.com/assets/Tech_Center/Ross_Broiler/Ross_Broiler_Manual_2014.

[38] Brody S. (1945). Bioenergetics and Growth, with Special Reference to the Efficiency Complex in Domestic Animals.

[39] McDonald P., Edwards R., Greenhalgh J., McDonald P., Edwards R.A., Greenhalgh J.F.D. (1973). Animal Nutrition.

[40] Belitz H.-D., Grosch W., Schieberle P. (2009). Meat. Food Chem..

[41] AOAC (2000). Official methods of analysis of AOAC International.

[42] Veterinary Medical Association (2013). AVMA Guidelines for the Euthanasia of Animals: 2013 Edition.

[43] Harrison G.J., Lightfoot T.L., Harrison L.R. (2006). Clinical Avian Medicine.

[44] Schalm O.W., Jain N.C., Carroll E.J. (1975). Veterinary Hematology.

[45] Trinder P. (1969). Determination of blood glucose using an oxidase-peroxidase system with a non-carcinogenic chromogen. J. Clin. Pathol..

[46] Henry R. (1974). Determination of serum creatinine. Clinical Chemistry: Principles and Techniques.

[47] Sanders G., Pasman A., Hoek F. (1980). Determination of uric acid with uricase and peroxidase. Clin. Chim. Acta.

[48] Reitman S., Frankel S. (1957). Determination of serum glutamic oxaloacetic transaminase and pyruvic transaminase by colorimetric method. Am. J. Clin. Path.

[49] Allain C.C., Poon L.S., Chan C.S., Richmond W., Fu P.C. (1974). Enzymatic determination of total serum cholesterol. Clin. Chem..

[50] McGowan M., Artiss J.D., Strandbergh D.R., Zak B. (1983). A peroxidase-coupled method for the colorimetric determination of serum triglycerides. Clin. Chem..

[51] Vassault A., Grafmeyer D., Naudin C., Dumont G., Bailly M., Henny J., Gerhardt M., Georges P. (1986). Protocole de validation de techniques. Ann Biol. Clin..

[52] Griffin H., Whitehead C. (1982). Plasma lipoprotein concentration as an indicator of fatness in broilers: Development and use of a simple assay for plasma very low density lipoproteins. Br. Poult. Sci..

[53] Grant G.H., Silverman L.M., Christenson R.H., Tietz N.Z. (1987). Amino acids and proteins. Fundamentals of Clinical Chemistry.

[54] Doumas B., Baysa D., Carter R., Peters T., Schaffer R. (1981). Determination of serum total protein. Clin. Chem..

[55] Doumas B.T., Biggs H.G., Arends R.L., Pinto P.V.C. (1972). Determination of Serum Albumin. Stand. Methods Clin. Chem..

[56] Rice-Evans C., Miller N.J. (1994). Total antioxidant status in plasma and body fluids. Methods Enzymol..

[57] Aebi H. (1984). Catalase in vitro. Methods in Enzymology.

[58] Nishikimi M., Rao N.A., Yagi K. (1972). The occurrence of superoxide anion in the reaction of reduced phenazine methosulfate and molecular oxygen. Biochem. Biophys. Res. Commun..

[59] Mcdonald R.E., Hultin H.O. (1987). Some characteristics of the enzymic lipid peroxidation system in the microsomal fraction of flounder skeletal muscle. J. Food Sci..

[60] Lie Ø., Syed M., Solbu H. (1986). Improved agar plate assays of bovine lysozyme and haemolytic complement activity. Acta Vet. Scand..

[61] Bancroft J.D., Gamble M. (2008). Theory and Practice of Histological Techniques.

[62] Saber S., Khalil R.M., Abdo W.S., Nassif D., El-Ahwany E. (2019). Olmesartan ameliorates chemically-induced ulcerative colitis in rats via modulating NFκB and Nrf-2/HO-1 signaling crosstalk. Toxicol. Appl. Pharmacol..

[63] Lipiński K., Mazur M., Antoszkiewicz Z., Purwin C. (2017). Polyphenols in monogastric nutrition–a review. Ann. Anim. Sci..

[64] Chamorro S., Viveros A., Centeno C., Romero C., Arija I., Brenes A. (2013). Effects of dietary grape seed extract on growth performance, amino acid digestibility and plasma lipids and mineral content in broiler chicks. Animal.

[65] Brenes A., Montoro A.V., Cambrodón I.G., Centeno C., Calixto F.S., Arija I. (2010). Effect grape seed extract on growth performance, protein and polyphenol digestibilities, and antioxidant activity in chickens. Span. J. Agric. Res..

[66] Chen C.-C., Hsu J.-D., Wang S.-F., Chiang H.-C., Yang M.-Y., Kao E.-S., Ho Y.-C., Wang C.-J. (2003). Hibiscus sabdariffa extract inhibits the development of atherosclerosis in cholesterol-fed rabbits. J. Agric. Food Chem..

[67] Yilmazer-Musa M., Griffith A.M., Michels A.J., Schneider E., Frei B. (2012). Grape seed and tea extracts and catechin 3-gallates are potent inhibitors of α-amylase and α-glucosidase activity. J. Agric. Food Chem..

[68] McDougall G.J., Shpiro F., Dobson P., Smith P., Blake A., Stewart D. (2005). Different polyphenolic components of soft fruits inhibit α-amylase and α-glucosidase. J. Agric. Food Chem..

[69] You Q., Chen F., Wang X., Luo P.G., Jiang Y. (2011). Inhibitory effects of muscadine anthocyanins on α-glucosidase and pancreatic lipase activities. J. Agric. Food Chem..

[70] Griffiths D.W. (1986). The inhibition of digestive enzymes by polyphenolic compounds. Nutritional and Toxicological Significance of Enzyme Inhibitors in Foods.

[71] Nasrawi M.A. (2013). Evaluation of Roselle Hibiscus sabdariffa flower as a nutritive additives on the productive performance of broiler. Iraqi J. Vet. Med..

[72] Hamodi S.J., Firas Al-M K. (2011). Compared between anise seeds (*Pimpinella anisum* L.) and Roselle flowers (hibiscussabdariffa) by their affected on production performance of broiler. Adv. Environ. Biol..

[73] Prommachart R., Cherdthong A., Navanukraw C., Pongdontri P., Taron W., Uriyapongson J., Uriyapongson S. (2021). Effect of Dietary Anthocyanin-Extracted Residue on Meat Oxidation and Fatty Acid Profile of Male Dairy Cattle. Animals.

[74] Villasante A., Patro B., Chew B., Becerra M., Wacyk J., Overturf K., Powell M.S., Hardy R.W. (2015). Dietary intake of purple corn extract reduces fat body content and improves antioxidant capacity and n-3 polyunsaturated fatty acid profile in plasma of rainbow trout, Oncorhynchus mykiss. J. World Aquac. Soc..

[75] Jaturasitha S., Ratanapradit P., Piawong W., Kreuzer M. (2016). Effects of feeding purple rice (*Oryza sativa* L. Var. Glutinosa) on the quality of pork and pork products. Asian-Australas. J. Anim. Sci..

[76] Kamboh A., Zhu W.-Y. (2013). Effect of increasing levels of bioflavonoids in broiler feed on plasma anti-oxidative potential, lipid metabolites, and fatty acid composition of meat. Poult. Sci..

[77] Munyaka P., Echeverry H., Yitbarek A., Camelo-Jaimes G., Sharif S., Guenter W., House J., Rodriguez-Lecompte J. (2012). Local and systemic innate immunity in broiler chickens supplemented with yeast-derived carbohydrates. Poult. Sci..

[78] Brenes A., Viveros A., Chamorro S., Arija I. (2016). Use of polyphenol-rich grape by-products in monogastric nutrition. A review. Anim. Feed Sci. Technol..

[79] Etxeberria U., Fernández-Quintela A., Milagro F.I., Aguirre L., Martínez J.A., Portillo M.P. (2013). Impact of polyphenols and polyphenol-rich dietary sources on gut microbiota composition. J. Agric. Food Chem..

[80] Hashemi S.R., Davoodi H. (2011). Herbal plants and their derivatives as growth and health promoters in animal nutrition. Vet. Res. Commun..

[81] Mitruka B.M., Rawnsley H.M. (1977). Clinical biochemical and hematological reference values in normal experimental animals. Clinical Biochemical and Hematological Reference Values in Normal Experimental Animals.

[82] Anon J. (1980). Guide to the Care and Use of Experimental Animal.

[83] Olagoke O.C., Akinwumi A.O., Emiola I.A. (2019). Growth Performance and Carcass Characteristics of Broiler Chicken Fed Diet Supplemented with Ginger (Zingiber Officinale), Garlic (Allium Sativum), Roselle (Hibiscus Sabdariffa) and their Combinations. Int. J. Res. Agric. Sci..

[84] Ugwu D., Jiwuba P., Ubogu V., Akazue R. (2020). Phytochemical properties of Hibiscus sabdariffa Calyx and the effects of its aqueous extract supplementation on haematological and serum biochemical indices of broiler birds. Niger. J. Anim. Sci..

[85] Asaniyan E., Akinduro V. (2020). Haematology and serum biochemistry of broiler chickens offered extracts of dried Roselle plant (*Hibiscus sabdariffa*) calyx in drinking water. Ife J. Sci..

[86] Islam M.R., Lepp D., Godfrey D.V., Orban S., Ross K., Delaquis P., Diarra M.S. (2019). Effects of wild blueberry (*Vaccinium angustifolium*) pomace feeding on gut microbiota and blood metabolites in free-range pastured broiler chickens. Poult. Sci..

[87] Azevedo J., Fernandes I., Faria A., Oliveira J., Fernandes A., de Freitas V., Mateus N. (2010). Antioxidant properties of anthocyanidins, anthocyanidin-3-glucosides and respective portisins. Food Chem..

[88] Yilmaz Y., Toledo R.T. (2004). Major flavonoids in grape seeds and skins: Antioxidant capacity of catechin, epicatechin, and gallic acid. J. Agric. Food Chem..

[89] Surai P. (2014). Polyphenol compounds in the chicken/animal diet: From the past to the future. J. Anim. Physiol. Anim. Nutr..

[90] Foti M., Piattelli M., Amico V., Ruberto G. (1994). Antioxidant activity of phenolic meroditerpenoids from marine algae. J. Photochem. Photobiol. B Biol..

[91] Wang Y., Ma X., Ye J., Zhang S., Chen Z., Jiang S. (2021). Effects of Dietary Supplementation with Bilberry Extract on Growth Performance, Immune Function, Antioxidant Capacity, and Meat Quality of Yellow-Feathered Chickens. Animals.

[92] Park I.-J., Cha S.-Y., Kang M., So Y.-S., Go H.-G., Mun S.-P., Ryu K.-S., Jang H.-K. (2011). Effect of proanthocyanidin-rich extract from Pinus radiata bark on immune response of specific-pathogen-free White Leghorn chickens. Poult. Sci..

[93] Schroeder H.W., Cavacini L. (2010). Structure and function of immunoglobulins. J. Allergy Clin. Immunol..

[94] Ardia D., Schat K. (2008). Ecoimmunology. Avian Immunology.

[95] Catoni C., Schaefer H.M., Peters A. (2008). Fruit for health: The effect of flavonoids on humoral immune response and food selection in a frugivorous bird. Funct. Ecol..

